# The interplay between infection risk factors of SARS-CoV-2 and mortality: a cross-sectional study from a cohort of long-term care nursing home residents

**DOI:** 10.1186/s12877-022-02779-0

**Published:** 2022-02-14

**Authors:** Laura Soldevila, Núria Prat, Miquel À. Mas, Mireia Massot, Ramón Miralles, Josep M. Bonet-Simó, Mar Isnard, Marta Expósito-Izquierdo, Irene Garcia-Sanchez, Sara Rodoreda-Noguerola, Nemesio Moreno, Esther Badia, Genís López, Javier Sevilla, Oriol Estrada, Xavier Vallès

**Affiliations:** 1 International Health Program, Regió Sanitària Metropolitana Nord, Institut Català de la Salut, Badalona, Spain; 2 Infectious Diseases Unit, Hospital Universitari Germans Trias i Pujol, Badalona, Spain; 3 Departament de Medicina, Universitat Autònoma de Barcelona, Bellaterra, Spain; 4 Fight AIDS and Infectious Diseases Foundation, Badalona, Spain; 5 Direcció d’Atenció Primària Metropolitana Nord, Institut Català de la Salut, Sabadell, Spain; 6 Direcció Clínica Territorial de Cronicitat Metropolitana Nord, Institut Català de la Salut, Badalona, Spain; 7 Department of Geriatrics, Hospital Universitari Germans Trias i Pujol, Badalona, Spain; 8 Institut per la Recerca en Ciències de la Salut Germans Trias i Pujol, Badalona, Spain

**Keywords:** Long-term care nursing homes, SARS-CoV-2, Epidemiology, Mortality, Covid-19

## Abstract

**Background:**

Covid-19 pandemic has particularly affected older people living in Long-term Care settings in terms of infection and mortality.

**Methods:**

We carried out a cross-sectional analysis within a cohort of Long-term care nursing home residents between March first and June thirty, 2020, who were ≥ 65 years old and on whom at least one PCR test was performed. Socio-demographic, comorbidities, and clinical data were recorded. Facility size and community incidence of SARS-CoV-2 were also considered. The outcomes of interest were infection (PCR positive) and death.

**Results:**

A total of 8021 residents were included from 168 facilities. Mean age was 86.4 years (SD = 7.4). Women represented 74.1%. SARS-CoV-2 infection was detected in 27.7% of participants, and the overall case fatality rate was 11.3% (24.9% among those with a positive PCR test). Epidemiological factors related to risk of infection were larger facility size (pooled aOR 1.73; *P* < .001), higher community incidence (pooled aOR 1.67, *P* = .04), leading to a higher risk than the clinical factor of low level of functional dependence (aOR 1.22, *P* = .03). Epidemiological risk factors associated with mortality were male gender (aOR 1.75; *P* < .001), age (pooled aOR 1.16; *P* < .001), and higher community incidence (pooled aOR 1.19, *P* = < 0.001) whereas clinical factors were low level of functional dependence (aOR 2.42, *P* < .001), Complex Chronic Condition (aOR 1.29, *P* < .001) and dementia (aOR 1.33, *P* <0.001). There was evidence of clustering for facility and health area when considering the risk of infection and mortality (*P* < .001).

**Conclusions:**

Our results suggest a complex interplay between structural and individual factors regarding Covid-19 infection and its impact on mortality in nursing-home residents.

## Introduction

It is estimated that in spite of representing less than five per cent of the elderly population (> 65 years old), 47% of all Covid-19 deaths occurred among residents from Long-Term Care Facilities (LTCF) nursing homes during the first wave of the SARS-CoV-2 pandemic in high-income countries [1]. This appalling data could be explained by the fact that LTCF show the ideal epidemiological conditions for transmission of an airborne infectious agent like SARS-CoV-2 since they are densely populated spaces with large numbers of staff who have extensive contact with highly vulnerable residents. The result has rightly been regarded as a perfect storm [2]. However, more detailed examinations of Covid-19 in LTCF have shown that particular structural factors mediated the impact of the disease, including the size of the facility, the quality of care, and understaffing [3–8]. The lack of preparedness as well as short and long-term policy failures also contributed [9, 10]. Indeed, besides the direct impact of Covid-19, there have been other secondary consequences due to the lockdowns and other measures intended to contain the infection, among the more important being the exacerbation of comorbidities and delayed medical treatment. Also, psychological, and emotional distress due to social isolation [11–14] and the lack of adequate end-of-life care [15], frequently reported during the first wave of the pandemic, added to the impact.

Most of the previous articles on the impact of Covid-19 in LTCF premises have explored risk factors, both individual [16] and structural [6], associated with mortality in LTCF residents. In previous articles, we analyzed contextual and clinical factors related to mortality of a local cohort during the first wave of Covid-19 in Europe [6, 17]. In this article we aim to expand previous analysis by focusing on epidemiological factors related to SARS-CoV-2 infection in LTCF and the interaction between contextual and societal factors in the evolution and results of SARS-CoV2 outbreaks and clinical prognosis.

## Methods

### Study population

The study region comprises an area immediately to the north of the city of Barcelona in Catalonia, Spain. Comprising a mixture of urban, semi-rural, and rural municipalities, the area includes a total of 1 986 032 inhabitants, with 190 LTCF which as of March first, 2020, housed a total of 10737 residents. Of these LTCF, 168 (88.4%) were institutions devoted to elderly adult patients and not linked to an hospital institution, with a total of 9553 users registered. Lockdown came into force at these institutions on March 15, 2020, and by the end of that month an emergency task force of health professionals had been mobilized to provide back-up support to LTCF staff.

### Study design

A cross-sectional study was carried out among a cohort study of residents of LTCF, defined as institutions which host for a long term people with age-related dependency and are not linked to an hospital institution,, living in the study area between first of March and June 30, 2020, who were ≥ 65 years old and on whom at last one PCR test was performed during the study period. Socio-demographic data (age and gender), and underlying chronic conditions as such as comorbidities, level of functional impairment and advanced conditions were recorded by their primary care teams according to local clinical guidelines [18], laboratory test results (specific Polymerase Chain Reaction henceforth PCR, to detect SARS-CoV-2) and clinical outcome (recovery/death), as well as the size of the LTCF (number of residents) and cumulative incidence of Covid-19 in the Primary Healthcare Catchment Area where the facility was located (this is the smallest administrative area of the Spanish public healthcare system, typically covering between 15 000 and 25 000 inhabitants attended by a primary health team). Following implemented guidelines, PCR testing was carried out when Covid-19 infection was suspected or when LTCF residents had come in close contact with an infected individual (i.e. when three or more confirmed or suspected cases of Covid-19 within the LTCF including staff members were detected). Regular testing was implemented starting in mid-April 2020 irrespective of infection suspicion or exposure. The outcomes of study were infection (PCR positive) and death.

### Data collection and statistical analysis

Data was entered in the database either by health staff using an in-house app developed *ad* hoc [19] or using the regional health service’s digital patient records (socio-demographic and clinical data), alongside PCR test results sent from the reference laboratory serving the region. Data regarding incidence from the catchment area was obtained from the official open-source database [20] and are expressed by cases over 10,000 inhabitants. Community incidence excluded all notified cases from LTCF located in the specific catchment area. LTCF capacity (number of residents) and community cumulative incidence were categorized according to interquartile ranges (IQR) of <25, 25-50, 50-75 and >75. PCR tests with inconclusive results were considered negative.

The data was analyzed using Stata v14.0 software (StataCorp, Texas) and R. For the descriptive analysis, we used means, medians, and Standard Deviation (SD) for continuous variables and proportions and a 95% Confidence Interval (95%CI) for categorical variables. Proportions were calculated for all participants with the given data available. For univariate analysis, we used the Chi-Square test to compare categorical variables, and the Student’s T test for continuous variables after testing for normality (skewness and kurtosis tests), or their non-parametric counterparts (Fisher test or Wilcoxon test), when necessary. Multilevel mixed-effects logistic regression was used for multivariate analysis to explore for the presence of clustering, and factors (LTCF and catchment area) and tested by means of regress post-estimation (likelihood ratio test, henceforth LR), against the usual logistic regression model. Crude and adjusted odds ratios (OR and aOR), respective 95%CI and *P* values were estimated.

### Laboratory methods (PCR test)

Biological specimens were collected through nasopharyngeal swabs. Specific PCR tests were carried out using the Aptima® SARS-CoV-2 assay following manufacturer instructions [21].

### Ethical approval

The study was approved by the Ethics Board of the reference hospital of the study region (Hospital Universitari Germans Trias i Pujol), registered under reference number PI-20-349, and was conducted in accordance with the principles of the Declaration of Helsinki.

## Results

### Participant characteristics

**Fig. 1:**
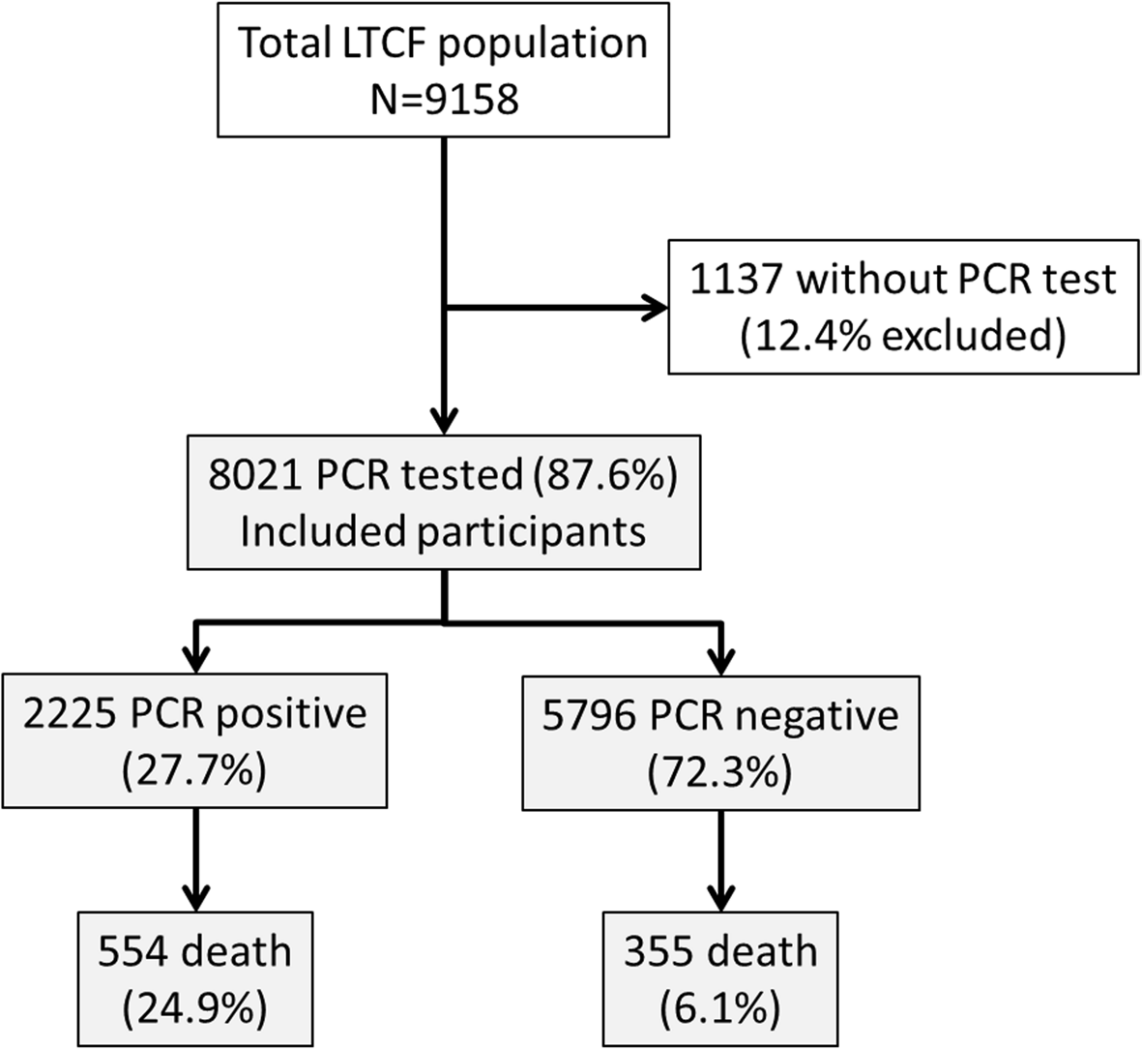
Flow-chart of study population and participants with PCR test available

**Fig. 2:**
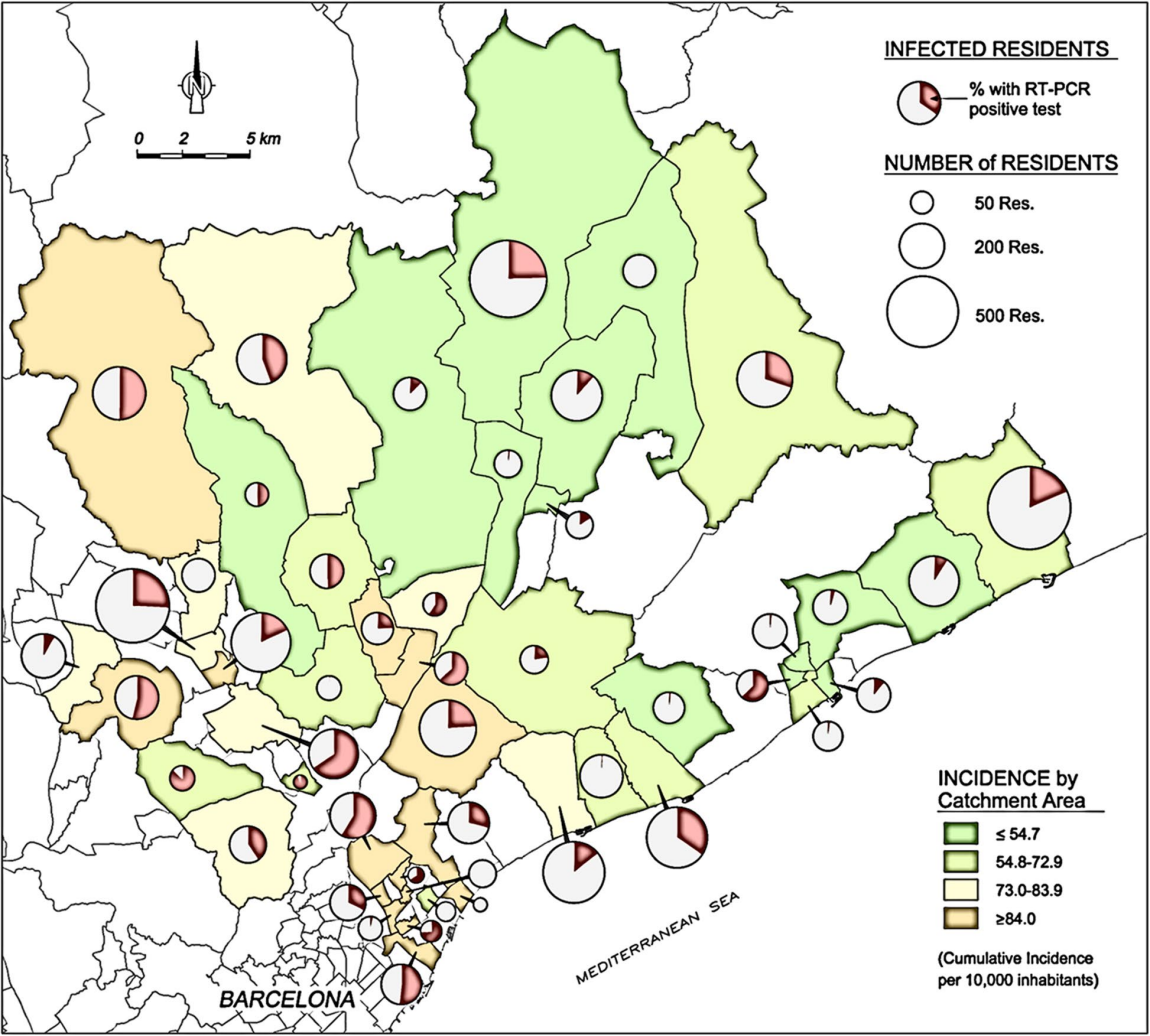
Cumulative incidence of SARS-CoV-2 infection of LTCLTCF residents and corresponding catchment area. The testing coverage was similar between different catchment areas during the study period and was mainly focused on symptomatic cases and contacts of positive cases. The incidence shown here is therefore an underestimation of the true incidence but should be considered proportional to it and an acceptable proxy. The size of the pie charts are correlated to the number of LTCF residents registered in each catchment area, not to the size of the LTCF’s

A total of 9158 residents ≥65 years residing in 168 LTCF were included in the study, with a median occupancy of 41 (range 12 to 229, IQR 26-41) and distributed over 52 local catchment areas. Of these residents, 8021 (87.6%) underwent at least one PCR test during the study period and were included in the analysis (see Fig. 1). Women accounted for 5939 (74.1%) and men for 2079 (25.9%). The mean age was 86.4 years (SD = 7.4, range 65-107), with significant difference between males (83.4 yrs., SD = 7.9) and females (87.4, SD = 6.9; *P* < .001). Overall, 1115 (14.0%) had a cardiovascular comorbidity. The most frequently recorded individual comorbidities were hypertension (n = 3855, 48.3%) and dementia (n = 3666, 45.9%). A total of 6013 patients presented functional impairment (80.3%) and 4171 (52.0%) an advanced condition. In the four-month study period, SARS-CoV-2 infection was detected in 2225 participants (27.7%), and 909 deaths were reported, of which 554 corresponded to residents with proven SARS-CoV-2 infection (Case Fatality Rate, henceforth CFR, 24.9%, see Fig. 1). At the LTCF level, 97 institutions (57.7%) reported at least one confirmed case of SARS-CoV-2 infection, with attack rates ranging from 1% to 95%. The median cumulative incidence of Covid-19 in the 52 catchment areas of the study region during the period of interest was 95 cases/10 000 inhabitants (range 34.7 to 158.2). Table 1 offers a description of the main variables in the total population, and in the subgroup of infected people and those that dye in the study period. Figure 2 shows the cumulative incidence of SARS-CoV-2 among residents and in catchment areas.

**Table 1:** Prevalence of study variables stratified by infection

	Total^a^		PCR positive			Death^b^		
	(N = 8021)		(N = 2225; 27.7%)			(N = 909; 11.3%)		
Variable	n	*%*	n	*%*	*P*	n	*%*	*P*
Gender								
Male	2079	*25.9*	597	*28.7*	.2	309	14.7	< .001
Female	5939	*74.1*	1626	*27.4*		600	10.1	
Age in years (m, SD)	86.4	*(7.4)*	86.5	*(7.3)*	.2	87.4	(7.1)	< .001
65-74	638	*8.0*	166	*26.0*	.2	47	5.2	
75-79	752	*9.4*	210	*27.9*		83	9.1	
80-84	1362	*17.0*	346	*25.4*		133	14.6	< .001†
85-89	2289	*28.6*	663	*29.0*		260	28.6	
≥90	2977	*37.1*	838	*28.2*		386	42.5	
Level of functional depdendence (low/high)^c^								
High level of functional dependence	6013	*80.3*	1497	*24.9*	< .001	357	5.9	< .001
Low level of functional dependence	1475	*19.7*	447	*30.3*		178	12.1	
Comorbidities								
Hypertension	3855	*48.3*	1164	*30.2*	< .001	488	12.7	.001
Diabetes Mellitus-II	1593	*20.0*	471	*29.6*	.08	208	13.1	.02
Chronic renal insufficiency	1564	*19.6*	451	*28.8*	.3	230	14.7	< .001
Dementia	3666	*45.9*	1002	*27.3*	.4	472	12.9	< .001
Cardiovascular disease ^d^	1115	*14.0*	363	*32.6*	< .001	170	15.3	< .001
Respiratory disease ^e^	824	*10.3*	262	*31.8*	.007	124	15.1	< .001
Cerebrovascular disease	211	*2.6*	46	*21.8*	.05	28	13.3	.4
Clinical complexity								
Complex Chronic Condition	723	*17.3*	202	*27.9*	.9	533	12.8	< .001
Number of residents ^f^								
≤ 40 (N = 84)	2060	*25.7*	1464	*17.6*	< .001	194	9.4	< .001^g^
41-72 (N = 44)	1986	*24.8*	1419	*22.3*		196	9.9	
73-108 (N = 25)	2049	*25.6*	1512	*31.0*		239	11.7	
> 108 (N = 16)	1926	*24.0*	1407	*40.7*		280	14.5	
Community cumulative incidence (cases by 10,000 inhabitants)								
≤ 54.7	1994	*25.4*	1811	*16.9*	< .001	137	6.9	< .001^g^
54.8-72.9	1903	*24.2*	1620	*28.6*		221	11.6	
73.0-83.9	1840	*23.4*	1515	*30.0*		200	10.9	
≥ 84.0	2115	*26.9*	1769	*36.8*		340	16.1	

^a ^Includes participants with data available

^b ^Includes mortality among participants with PCR test result available

^c ^Levels of functional dependence have been calculated using the Barthel score, which provides a range from 0 to 100. The cut-off between high and low functional dependence has been set up at 50

^d ^Includes ischemic heart disease and heart insufficiency

^e ^Include Chronic Obstructive Pulmonary disease, asthma, and emphysema

f N indicates the number of LTCF institutions included in each strata

^g ^*P* test for trend

### Risk factors associated to SARS-CoV-2 infection and death

**Fig. 3:**
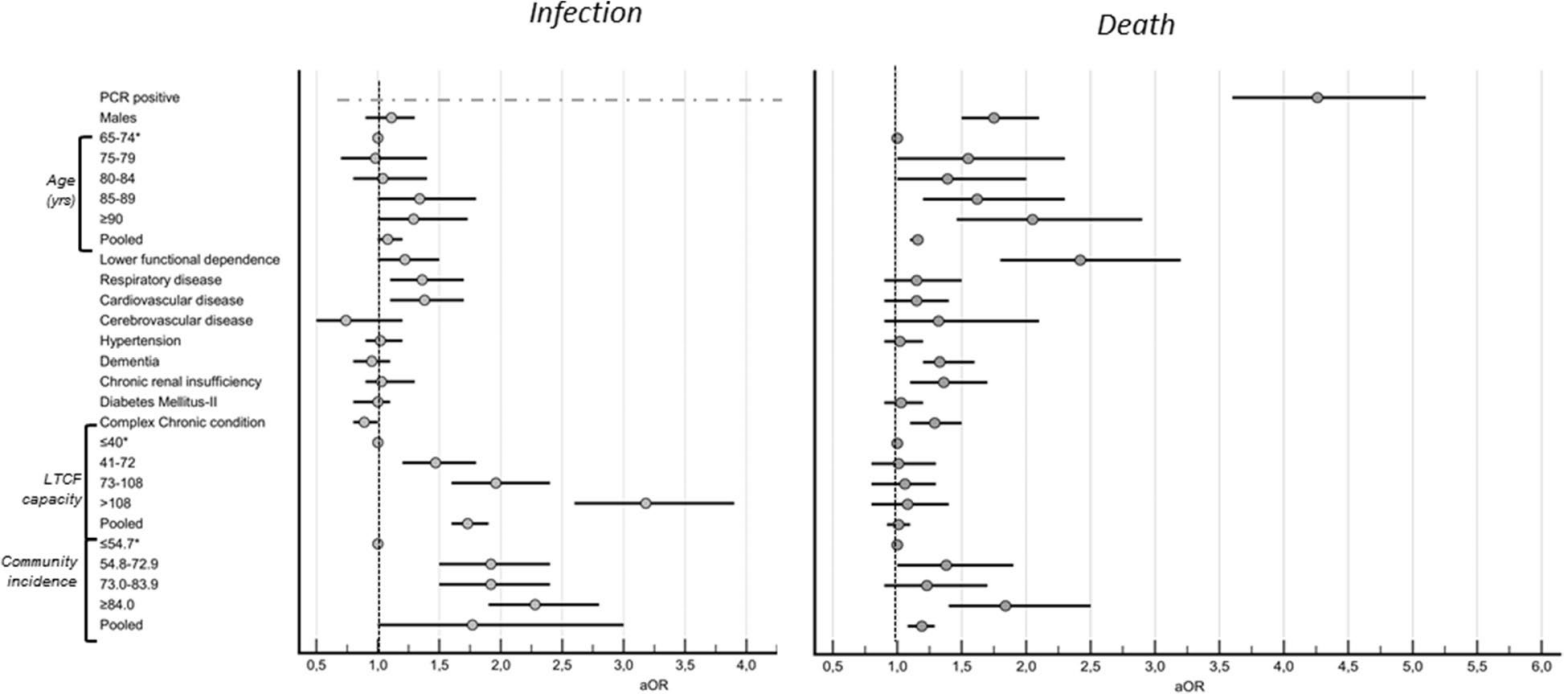
Forest plot of a OR and 95%CI of studied variables and infection (PCR positive) and death. §. Baseline strata. *Significative results (*p* ≤ 0,05)

Figure 3 displays the aOR of study variables with SARS-CoV-2 infection and death in our analysis. In brief, the risk of infection by SARS-CoV-2 was independent of age, sex, and chronic conditions, except respiratory diseases and cardiovascular disease (*P* values of .01 and .005, respectively) and low level of functional dependence on daily living (low level of functional dependence score, aOR 1.22; 95%CI 1.0-1.5; *P* = .03). There was a linear correlation between the risk of infection and size of LTCF (pooled aOR 1.73; 95%CI 1.6-1.9; *P* < .001) and cumulative incidence in the catchment area (pooled aOR 1.77; 95%CI 1.0-3.0; *P* = .04). We observed evidence of clustering by LTCF and catchment area regarding risk of infection (*P* value of LR < .001). After adjusting for study variables, epidemiological risk factors related to infection that remain associated with mortality included SARS-CoV-2 infection (positive PCR test, aOR 4.26; 95%CI 3.6-5.1; *P* < .001), followed by low functional dependence (aOR 2.42; 95%CI 1.8-3.2; *P* < .001), male gender (aOR 1.75; 95%CI 1.5-2.1; *P* < .001) and older age (pooled aOR 1.16; 95%CI 1.1-1.2; *P* < .001), whereas dementia and Complex Chronic Condition was associated to mortality (aOR 1.33; 95%CI 1.2-1.6; *P <* .001 and aOR 1.29; 95%CI 1.1-1.5;* P* < .001, respectively) but not to infection. We observed evidence of clustering regarding mortality with LTCF and catchment area (*P* value of LR < .001). Furthermore, residents living in nursing homes located in catchment areas with a high incidence of SARS-CoV-2 (≥ 84/10 000 inhabitants) were at substantially higher risk of mortality (pooled aOR 1.19; 95%CI 1.1-1.3; *P* < .001).

## Discussion

Our results indicate that there is a complex interplay between the risk of SARS-CoV-2 infection and subsequent clinical outcome, mediated by individual level factors (age, gender, and chronic conditions) and contextual factors. Particularly, size of residence and community incidence showed the highest influence on infection acquisition, as has been observed previously [6, 22], with high OR (3.18 and 2.28, respectively). This may be explained because large nursing homes were more susceptible to a SARS-CoV-2 carrier entering the premises due to the higher number of visiting relatives and working staff. Consequently, larger facilities had a greater likelihood of having one or more cases of Covid-19 compared to smaller ones; (88.1% vs. 37.0%, P < .001). However, our data showed highly heterogeneous attack rates once the infection was inside an institution (from 1% to 95%). This may be related to structural factors of each LTCF, which showed disparate features (including quality of the service provided and residents’ clinical profile), as observed in a previous study involving this study population [6] and a comprehensive study carried out in England [8]. This is consistent with the clustering effect seen here. The correlation between community incidence and risk of infection in LTCF has been described previously [23]. At first glance, the straight correlation may reflect higher chances of irruption in LTCF of infected individuals from the general population which include LTCF staff and visiting relatives before the lockdown. However, it cannot be ruled out that during the first wave and before the lockdown, these institutions could have become, to a certain extent, hubs of SARS-CoV-2 infection in the community. This would have been similar to the role that community health centers played during the 2014-2016 Ebola crisis in West Africa [24, 25]. LTCF staff are known to have been heavily infected whilst at work [26, 27], prior to the implementation of effective contention measures, and most probably also visiting relatives before the lockdown, and to have spread the infection within the local communities specially in crowded, urban and densely populated areas. If this hypothesis turns out to be true, the rapid intervention in LTCF carried out by the health authorities’ right at the outset of the Covid-19 pandemic was more relevant than supposed. It is worth noting that we do not consider this to be currently the case due to the strict Covid-19 prevention measures that have been applied in LTCF since mid of March (strict lockdown, interdiction of leaving the premises and visits, generic non-pharmaceutical measures and the hand-over of the LTCF clinical management to the Primary Health care teams which occurred on 10 of April). Moreover, the correlation between mortality and community incidence has been observed elsewhere and by our group in a previous analysis [6] and deserves further assessment using the LTCF setting as unit of analysis.

Focusing on gender, we found that being male is not correlated to SARs-CoV-2 infection but it is an independent risk factor for severe Covid-19 disease, as previously observed among general and elderly populations [16, 28]. The underlying reasons have not yet been disentangled, but this strongly suggests some genetic-based susceptibility [29]. In spite of this, considering the disproportion between males/females (25.9% vs. 74.1%), females showed a much higher absolute number of fatalities (600 vs. 309). This cannot simply be explained by the longer life expectancy of women but must also reflect societal gender-based inequalities that increase the likelihood of females ending up in a LTCF compared to males. Given the disproportionate contribution of LTCF residents to the global rate of Covid-19 deaths [1], the observed overrepresentation of female mortality in Spain [30] may be explained by, first, an increased proportion of females in Spanish LTCF compared to other countries and, second, the greater impact of the first wave of Covid-19 in LTCF observed in this country [30].

Besides these factors, we observed an intriguing interplay between risk of infection and mortality. More autonomous residents (low level of functional dependence) showed a notably higher risk of SARS-CoV-2 infection, but a disproportionate mortality rate as well, as we published previously [17]. This may be explained by the fact that residents with greater autonomy may have had a higher rate of social contacts inside the LTCF and therefore a higher probability of exposure to infection. The higher risk of mortality once infected may be a consequence of more efficient transmission and/or multiple infections (closeness to and increased frequency of risky contacts) with associated to higher viral loads, which has been correlated with mortality [31]. This association between risk of infection and autonomy as measured by Activities of Daily Living scores has been previously observed but not discussed [3]. The increased risk of infection associated with cardiovascular and respiratory disease could easily be biased in a population with a high prevalence of such conditions. According other reports, besides chronic renal failure [17], we observed a lack of association between negative outcome and other underlying comorbidities described among general populations [28, 32, 33], and patients with Covid-19 in geriatric care [34]. We hypothesized that in the context of extremely high attack rates in LTCF, once SARS-CoV-2 infection occurs tends to behave independently of underlying risk factors, like a primary pathogen. Furthermore, existing medications may have had a substantial effect against infection or severe disease in a population that is usually polymedicated [35].

Our study has some limitations. A substantial number of residents did not undergo PCR testing (n = 1154; 12.6%) because it was not possible to mobilize enough material and human resources in time amidst the extremely demanding circumstances of the early stages of the epidemic wave, and only later on was general screening implemented (firstly, only symptomatic cases were screened, in later stages all exposed residents and finally regular mass screenings were implemented). Our cross-sectional approach relies under the assumption that almost all residents had similar chances to be tested during the study period. As in other reports, our data indicate that many deaths occurred in people who were infected with Covid-19 but not tested [36]. The CFR of this sample subset (those that did not undergo a PCR test) was 69.2% (n = 798, see Fig. 1), which increases the death toll rate of our study population to 18.6%. This toll is close to the expected total mortality rate among our cohort for an entire year [37]. Considering that most fatalities were Covid-19 patients, the specific CFR among SARS-CoV-2-infected residents was around 50%. This estimate lies in the upper bounds of previous estimates [1, 27, 38]. Therefore, because it included only patients for whom a PCR test was available and thus possibly excludes the most vulnerable patients, our analysis could be to a certain extent biased. Non-tested residents tended to be older (87.4 years vs. 86.4, *p* < .001), although the gender distribution was similar (*p* = .2). Nonetheless, the lower mortality rate observed in later stages may reflect the effectiveness of the contention measures implemented. Otherwise, a number of variables that may influence on the risk of infection (i.e. proximity between residents or number of staff who tested positive) have not been considered in this study but were extensively reported in the referenced work in the same study population [6].

Overall, our results may explain the much higher CFR (around 40%) of older adults living in LTCF infected by SARS-CoV-2 compared to the general CFR observed in people older than 80 years old, estimated to be 14.5% [39]. due to the interaction between of structural (institutional), as the clustering of our data suggests, which were in turn consequence of societal and implemented policies, and individual factors (related to chronic/advanced conditions, age, gender and autonomy level, as previously discussed [17], which may be more or less prevalent in each facility. However, it is worth noting that these results correspond to the conditions of the first wave of a pandemic, which tends to be characterized by a lack of preparedness against an unknown pathogen. Therefore, other risk factors correlated to infection and negative outcome in LTCF may emerge in later stages of the pandemic.

In spite of the overwhelmingly positive impact of the vaccination of LTCF residents [40, 41], given the current uncertainty about the role of vaccines in preventing transmission in nursing homes at mid and long term, as well as the impact of new variants of the virus [42], research on Covid-19 in LTCF facilities should still be prioritized. The scope of research Covid-19 in nursing homes should be expanded, as well, to ascertain the indirect consequences of not only lockdown measures but also measures intended to mitigate these effects (i.e. to safely allow family visits) [43–45] as well as the ways by which such measures might be avoided in the future [46] Furthermore, long-term policies should address structural factors underlined by our results and previous publications, to create a more adequate elderly care system like the prioritization of home-based care, the promotion of affordable smaller-scale, high-quality group models with a community-based approach for those that caregiving at home is not feasible, and a more comprehensive and non-profit orientated regulations [47].

## Conclusions

There was a complex interplay between structural and individual factors regarding Covid-19 infection and its impact on mortality in nursing-home residents during the first epidemic wave. The overwhelming impact on LTCF residents could be partially explained by the intersect of lack of preparedness and structural factors. Risk of SARS-CoV-2 infection was mainly associated to contextual level factors (i.e. LTCF structure), whereas risk of mortality to individual level factors (i.e. frailty). Long-term policies should be implemented to develop a residential system more resilient to epidemic surges. Secondary effects due to lockdowns and social restrictions among elderly residents should be investigated.

## Data Availability

The data underlying this article cannot be shared publicly due to it is subject to Ethical approval before accessing to it. The data will be shared on reasonable request to the corresponding author and with previous Ethical Approval by the Ethics Board of reference.
